# Macroscopic and Microscopic Performance Study of Filling-Type Large-Size Cement-Stabilized Macadam

**DOI:** 10.3390/ma18245501

**Published:** 2025-12-07

**Authors:** Jin Ran, Hailin Wang, Dong Tang, Naitian Zhang, Meiling Li, Yanshun Jia, Lianxia Ma, Yinbo Zhang

**Affiliations:** 1 Xinjiang Key Laboratory of Green Construction and Smart Traffic Control of Transportation Infrastructure, Xinjiang University, Urumqi 830017, China; ranjin@xju.edu.cn (J.R.); znt@xju.edu.cn (N.Z.); 2 School of Traffic and Transportation Engineering, Xinjiang University, Urumqi 830017, China; wanghailin@stu.xju.edu.cn; 3 School of Traffic Engineering, Shandong Jianzhu University, Jinan 250101, China; limeiling2015@hotmail.com; 4 School of Traffic and Transportation, Shijiazhuang Tiedao University, Shijiazhuang 050043, China; jiaysh@stdu.edu.cn; 5 Xinjiang Transportation Construction Group Co., Ltd., Urumqi 830000, China; malianxia@xjjjjt.com (L.M.); zhangyinbo@xjjjjt.com (Y.Z.); 6 School of Ecology and Environment, Xinjiang University, Urumqi 830017, China

**Keywords:** filling-type large-size cement-stabilized macadam, interfacial transition zone, nanoindentation, micromechanical properties, shrinkage resistance, fatigue performance, macroscopic mechanical behavior

## Abstract

**Highlights:**

**What are the main findings?**

- F-LSBC exhibits 60–75% ITZ modulus and 55% hardness compared to conventional CSM.
- The ITZ of F-LSBC is thicker (55–90 μm) and 1.5× larger in volume fraction than in CSM.
- F-LSBC shows lower strength and fatigue life due to pronounced interfacial weakening.
- High coarse aggregate content and weak interface reduce overall shrinkage deformation.
- Nanoindentation–macro testing links ITZ microstructure to macroscopic performance.

**What are the implications of the main findings?**

- Interface engineering is key to balancing strength and crack resistance in F-LSBC bases.
- Weak ITZ design provides “crack-without-displacement” behavior for shrinkage control.
- Enhancing ITZ compactness can improve both mechanical durability and fatigue resistance.
- Results guide optimization of semi-rigid base materials for reflection crack mitigation.
- Findings support durable, low-shrinkage cement-stabilized base design for pavements.

**Abstract:**

Filling-type large-size cement-stabilized macadam (F-LSBC) is a promising base material for mitigating reflection cracking in semi-rigid pavements. However, its engineering application is hindered by the challenge of balancing strength, crack resistance, and construction adaptability. More fundamentally, the relationship between micromechanical features—especially the interfacial transition zone (ITZ)—and the macroscopic behavior of filling-type cement-stabilized composites remains insufficiently understood. This study used conventional cement-stabilized macadam (CSM) as a reference and combined nanoindentation with macro-scale mechanical, fatigue, and drying shrinkage tests to clarify the micro–macro mechanisms of F-LSBC. Results show that the ITZ in F-LSBC exhibits substantially lower elastic modulus (reduced by 60–75%) and hardness (reduced by 55%), along with greater porosity and phase volume fraction than CSM. Cluster analysis revealed a thicker ITZ (55–90 μm vs. 40 μm), indicating notable interfacial weakening. These microstructural features lead to reduced strength and fatigue life. Nevertheless, due to its high coarse aggregate content and weak-interface-induced “crack-without-displacement” mechanism, F-LSBC demonstrates enhanced shrinkage resistance, with drying shrinkage reduced to 81.36% of that of CSM at 180 days. The findings emphasize the key role of ITZ characteristics in determining performance and suggest that improved interface engineering could enhance durability and shrinkage control in pavement bases.

## 1. Introduction

Cement-stabilized macadam (CSM) base has been widely adopted in semi-rigid asphalt pavement structures due to its excellent load-bearing capacity, low elastic deformation, and relatively low cost [1,2]. As a key load-bearing layer, CSM plays a fundamental role in supporting the pavement’s overall structural performance [3]. However, under long-term service conditions, CSM is prone to fatigue damage caused by repeated traffic loading. In addition, its high shrinkage sensitivity makes it susceptible to shrinkage cracking under fluctuating temperature and humidity. These cracks not only compromise the integrity of the base layer but also tend to propagate upward under sustained loads, ultimately causing through-cracks in the asphalt surface—commonly referred to as reflection cracks [4,5]. Reflection cracks disrupt structural continuity and compaction of the pavement, allowing moisture to penetrate and accelerating structural deterioration. As a result, service life is significantly reduced, and maintenance frequency and associated costs are substantially increased [6].

Various mitigation strategies have been proposed to address reflective cracking, which can generally be categorized into five types: (1) direct crack repair, such as crack sealing or polymer grouting [7]; (2) surface layer optimization, including increasing structural thickness [8], applying high-performance modified materials [9], or employing thermal recycling techniques [10]; (3) installation of interlayer crack-resistant systems, such as graded aggregate bases, polyester geotextiles, or stress-absorbing rubberized asphalt layers [11,12,13]; (4) material-level improvements aimed at enhancing the performance of the base layer [14,15,16]; and (5) activation treatments applied to crushed stone, such as alkali activation [17,18,19] or microbial activation [20,21], which modify the aggregate surface properties and improve its compatibility with cementitious binders. The first three strategies primarily intervene at the crack-propagation stage or address its visible manifestations. While they can delay the onset of reflective cracking to some extent, they fail to address the underlying causes at the material level. The fourth and fifth categories provide more fundamental improvements at the material level; however, the fifth category often involves relatively complex processing routes and higher costs, which may limit its large-scale engineering application. In contrast, improving resistance to drying shrinkage and cracking through optimized material composition and structural design at the base level is considered the most effective way to control reflective cracking at its origin. Therefore, developing new base materials with superior cracking resistance, while maintaining acceptable construction complexity and cost, is crucial for improving pavement durability and extending service life.

Against this background, the development of filling-type large-size cement-stabilized macadam (F-LSBC) emerged. This material was proposed by the Chuzhou Highway Administration Bureau of Anhui Province in 2004, based on extensive experience in long-term reconstruction of existing roads. It was first applied in 2005 in the reconstruction project of Chuliang Road (X003) in Chuzhou [22]. Due to its outstanding performance in crack resistance, F-LSBC has been gradually promoted nationwide since 2015. In view of its remarkable advantages in mitigating reflection cracking, the national Technical Specification for Large Stone Base Course Filled with Cement Stabilized Macadam (T/CECS G: K23-01-2019) [23] was issued in February 2019 and officially implemented in July of the same year, providing a unified technical standard and design basis for its engineering application.

The design concept of F-LSBC is to construct an interlocked skeleton structure using oversized coarse aggregates, with fine aggregates (fillers) filling the voids between the coarse particles. This approach aims to achieve a skeleton-dense structure, thereby enhancing the material’s overall stability and crack resistance [24,25]. Within this design framework, the extended contact time between cement and fine aggregates leads to the concentration of hydration products primarily in the binder–filler matrix, while the interface between coarse aggregates and the filler forms a relatively loose interfacial transition zone (ITZ) that exhibits characteristics of a “weak interface.” Recent advances in ITZ micromechanics have demonstrated that the mechanical weakness of the ITZ is closely related to its heterogeneous microstructure, including elevated porosity, dispersed low-density calcium–silicate–hydrate (C–S–H), and the presence of microcracks formed during early age shrinkage [26]. Nanoindentation-based phase quantification has further revealed that the ITZ typically contains a higher proportion of low-modulus phases and less densified C–S–H compared with the bulk matrix, resulting in lower elastic modulus and hardness [27]. These findings not only support the conceptualization of the ITZ as a mechanically “weak interface” but also highlight its critical role in governing local stress transfer and shrinkage-induced deformation in cement-stabilized materials. Integrating these insights into the F-LSBC structure design, the intentionally weakened ITZ provides additional deformation freedom, enabling the dissipation of shrinkage-induced strain energy and thereby improving the crack-resistance capability of the entire composite system.

The core objective of F-LSBC is to constrain the shrinkage of the filler using the coarse aggregate skeleton, while simultaneously allowing for the release of shrinkage-induced strain energy through the weak interface. Although a weak interface may reduce the material’s overall strength and modulus, the dense skeleton structure effectively mitigates these effects. Notably, this structural mechanism is similar to the “preset microcrack network” approach used to control shrinkage cracking, in that both rely on the release of strain energy to reduce stress concentrations. The key difference lies in the formation of microcracks: in F-LSBC, interfacial weakening naturally induces more uniformly distributed and larger-scale microcracks, which result in a greater capacity for strain energy dissipation. Engineering practice has demonstrated that F-LSBC offers significant advantages in mitigating reflection cracking, and in recent years, it has gained recognition as a promising solution to the persistent issue of reflective cracking in semi-rigid base asphalt pavements.

Wang et al. proposed a mix proportion design for filling-type stone base courses based on the concept of enhanced crack resistance [28]. Building on this, Pan et al. employed a range of microscopic testing techniques to investigate the crack-resistance mechanism of F-LSBC at the microscale [29]. Li further advanced the field by analyzing the formation mechanism of reflection cracks in semi-rigid bases from an energy-evolution perspective and, accordingly, proposed a skeleton-dense F-LSBC design strategy rooted in the intentional weakening of the ITZ around coarse aggregates [30]. On this basis, Xia et al. optimized the F-LSBC mix design to improve its overall strength [31]. Despite the above advancements, three important research gaps remain unaddressed in the existing literature. First, previous studies have not quantitatively characterized the ITZ in F-LSBC in terms of micromechanical phase distribution, volume fraction, and porosity, leaving the actual degree of interface weakening insufficiently validated. Second, there is a lack of integrated micro–macro evaluation frameworks that combine nanoindentation-based micromechanics with macroscopic strength, fatigue, and shrinkage behaviors, limiting the ability to establish mechanistic correlations across scales. Third, existing ITZ evaluations rarely incorporate porosity back-calculation or statistically deconvoluted indentation phase analysis, both of which are essential for accurately assessing the deformation and failure mechanisms of weak interfaces in semi-rigid base materials.

To address these gaps, the present study makes several key contributions. First, we deliver a rigorous quantitative characterization of the ITZ in F-LSBC—encompassing phase classification, elastic modulus distribution, and ITZ volume fraction—using high-resolution nanoindentation mapping coupled with cluster analysis. Second, we develop an integrated micro–macro evaluation framework that systematically links micromechanical features with the macroscopic mechanical, fatigue, and drying shrinkage behaviors of F-LSBC, thereby enabling a more mechanistic interpretation of its crack-resistant response. Third, porosity back-calculation and ITZ-specific micromechanical indicators are introduced to more accurately elucidate how interface weakening facilitates strain accommodation and mitigates reflection-crack susceptibility. Taken together, these contributions advance the fundamental understanding of the deformation mechanisms governing filling-type structures and offer a robust scientific basis for improving the engineering applicability of F-LSBC.

## 2. Materials and Methods

**Figure 1:**
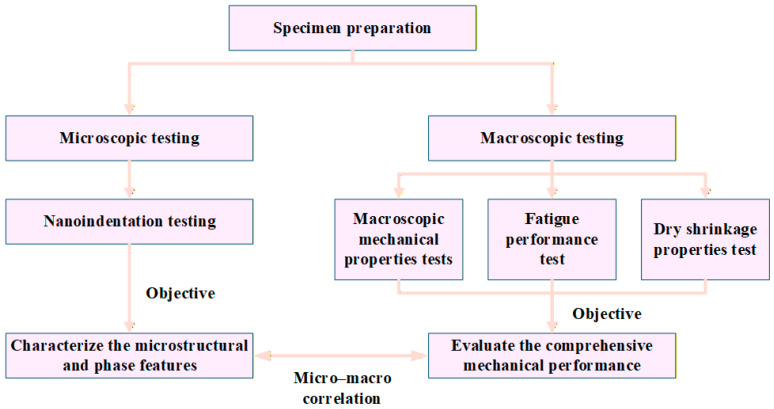
Experimental flowchart.

To improve the clarity of the experimental plan, a workflow diagram summarizing the entire research process is added at the beginning of this section (see Figure 1). The flowchart outlines the sequential steps, including material preparation, specimen fabrication, nanoindentation testing and statistical analysis, macroscopic mechanical testing, fatigue evaluation, and drying shrinkage measurements. This visual structure helps readers clearly understand the relationship between different experimental stages and the overall methodology framework.

### 2.1. Specimen Preparation

#### 2.1.1. Synthetic Gradation

**Figure 2:**
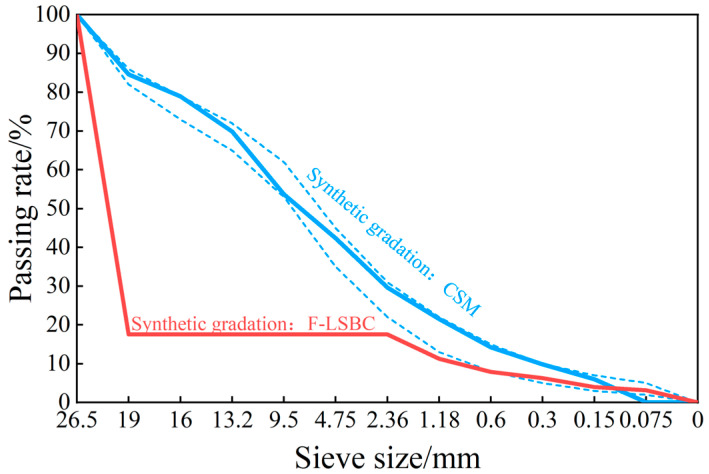
The synthetic gradation of F-LSBC and CSM. The synthetic gradation curves of filling-type large-size cement-stabilized macadam (F-LSBC) and con-ventional cement-stabilized macadam (CSM), where the interrupted blue line corresponds to the gradation of F-LSBC.

Both F-LSBC and CSM utilized ordinary Portland cement with a strength grade of P.O 42.5, and the specific technical parameters are provided in Table 1. For F-LSBC, coarse aggregate consisted of limestone with particle sizes ranging from 19 to 26.5 mm, while fine aggregate was limestone with a maximum particle size of 2.36 mm. The mass of fine aggregate was set at 0.213 times that of the coarse aggregate [22]. The resulting synthetic gradation of F-LSBC based on this proportion is detailed in Table 2 and illustrated in Figure 2. As for CSM, three types of limestone aggregates with different particle sizes—designated as 1#, 2#, and 3# (arranged from largest to smallest)—were used. Its synthetic gradation was determined based on the skeleton dense gradation range recommended in the specification JTG/T F20-2015 [24], and the results are likewise presented in Table 2 and Figure 2.

#### 2.1.2. Physical Parameters

According to existing literature, the cement content (c%) of the filler in F-LSBC was determined to be 30%, under which the maximum dry density of the filler reached 2.240 g/cm^3^, and the optimum moisture content was 8.27%. For the overall F-LSBC material, the corresponding cement content (c’%) was 5.27%, with a moisture content of 1.79% and a dry density of 2.294 g/cm^3^ [30]. To facilitate a direct comparison with F-LSBC, the cement content (c’%) for CSM was also set at 5.27%. After determining the synthetic gradation and cement dosage for CSM, the maximum dry density $\rho_{dry}^{max}$ and optimum moisture content $w_{OP}$ were further obtained through compaction tests. Additionally, the coarse aggregate intergranular void ratio ${VCA}_{WCC}$ and total void ratio ${VV}_{WCC}$ under the wet compaction state were calculated with reference to [30], and the specific results are presented in Table 3.

#### 2.1.3. Specimen Preparation and Curing

This study conducted performance tests on F-LSBC and CSM using two specimen types: cylindrical and beam-shaped. Both types were compacted using a static press under controlled conditions to achieve 100% compaction. Given that the nominal maximum particle size of both materials is 26.5 mm—categorizing them as coarse-grained soils—large-sized specimens were required for testing. The cylindrical specimens had dimensions of (Φ 150 mm×H 150 mm), while the beam specimens were selected as medium beams, with dimensions of (l 400 mm×b 100 mm×h 100 mm), in accordance with specification JTG E51-2009 [32], which permits a nominal maximum particle size of 26.5 mm. Forming large beams is impractical.

After specimen preparation, both cylindrical and beam specimens were cured in a curing chamber until the designated age, following the requirements of specification JTG E51-2009 [32]. Two curing methods were employed: standard curing and accelerated curing, both conducted at a relative humidity of at least 95%. The standard curing temperature was maintained at 20 ± 2 °C, while the accelerated curing temperature was set at 60 ± 1 °C. Specifically, standard-cured specimens were transferred to a 20 ± 2 °C constant-temperature water bath for saturation on the final day of the curing period. For accelerated curing, the specimens were removed from the high-temperature chamber on the final day, cooled at room temperature for 2 h and then transferred to the 20 ± 2 °C water bath for saturation treatment.

### 2.2. Microscopic Testing

#### 2.2.1. Principle of Nanoindentation Testing

**Figure 3:**
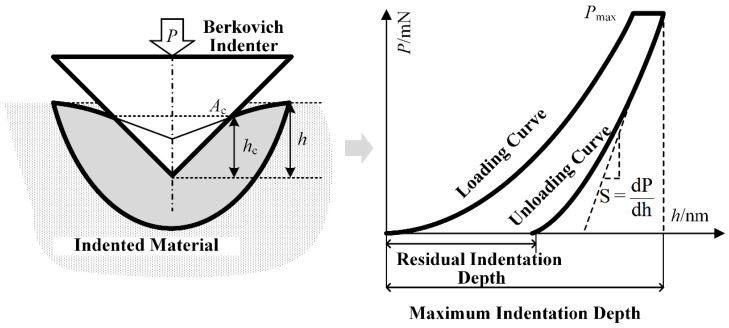
Principle of nanoindentation testing.

The basic principle of nanoindentation testing is to press a sharp indenter into the surface of a material. After maintaining a constant load for a specified duration, the load is unloaded while continuously recording the applied load (P) and the corresponding displacement (h). This process yields a load–displacement (P–h) curve, as illustrated in Figure 3. Based on this curve, the indentation hardness and the elastic modulus of the material in the indentation region can be further calculated according to references [33,34].

(1) $$H=\frac{P_{max}}{A_{c}}$$

(2) $$E_{r}=\frac{\sqrt{\pi }}{2\beta }˙\frac{S}{\sqrt{A_{c}}}$$

(3) $$E=\frac{1-v^{2}}{\frac{1}{E_{r}}-\frac{1-v_{i}^{2}}{E_{i}}}$$

where H is the indentation hardness (GPa), and $E_{r}$ is the indentation modulus (GPa). $P_{max}$ represents the maximum load applied at the indentation point (mN), and $A_{c}$ is the projected contact area under the maximum load. S denotes the contact stiffness (GPa), and β is a correction factor for the indenter geometry. E and v are the elastic modulus (GPa) and Poisson’s ratio of the tested material at the indentation site, respectively, with v typically taken as 0.3. $E_{i}$ and $v_{i}$ represent the elastic modulus and Poisson’s ratio of the indenter material, respectively. For the most commonly used diamond indenter, $E_{i}$ is 1141 GPa, and $v_{i}$ is 0.07.

**Figure 4:**
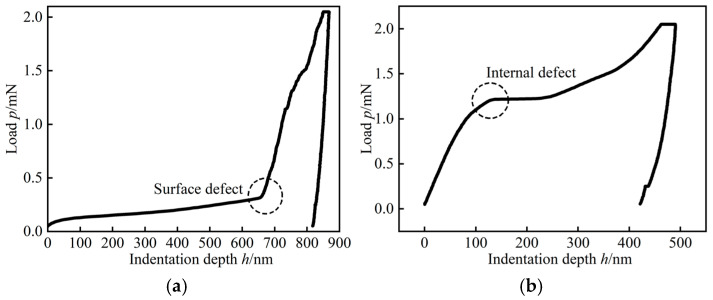
Nanoindentation test results: abnormal P–h curves: (**a**) Surface defect at the indentation site. (**b**) Internal defect at the indentation site.

For typical multiphase heterogeneous materials such as F-LSBC and CSM, the presence of initial defects—such as microcracks—is inevitable within the specimens. As a result, some of the nanoindentation load–displacement (P–h) curves exhibit distinct non-ideal characteristics, deviating from the expected continuous and smooth profile, as shown in Figure 4. Specifically, when the indentation region coincides with a surface defect, the load increases slowly in the initial stage, followed by a sudden rise at a certain depth, producing a distinct inflection point (Figure 4a). If the defect is located within the interior of the material, the load increases rapidly at first and then shows a significant reduction in growth rate at a certain depth, or even a local drop in load (Figure 4b). Although these abnormal curves may reflect local discontinuities or damage characteristics within the material, they deviate from the true mechanical response and cannot accurately represent the intrinsic mechanical properties. Therefore, such data must be excluded from the analysis to ensure the reliability of the evaluated indentation hardness and modulus.

During the experiment, the indentation hardness (*H*) and the indentation modulus ($E_{r}$) were automatically calculated by the instrument, whereas the elastic modulus (*E*) needed to be manually calculated in subsequent analysis using Equation (3). In this study, indentation hardness (*H*) and elastic modulus (*E*) were adopted as representative micromechanical properties for further analysis.

#### 2.2.2. Statistical Nanoindentation Principle

Nanoindentation technology was initially developed for single-phase homogeneous materials and was later extended to multiphase heterogeneous materials such as cement-based systems. However, this extension introduced the so-called “hybrid effect,” wherein the mutual interference among different phases in multiphase materials makes it difficult to accurately determine the elastic modulus or hardness of an individual phase. To address this issue, the Uim research group at the Massachusetts Institute of Technology proposed a statistical nanoindentation technique in 2003, specifically tailored for multiphase materials [35,36,37]. This method is based on the following theoretical assumption: if a multiphase material consists of two phases and the characteristic particle size of each phase is significantly larger than the indentation depth, then each indentation point represents the properties of a single phase, unaffected by the other phase. Consequently, the probability of a particular phase being indented corresponds to its area fraction within the testing region [38].

In practice, the elastic modulus (or hardness) values at all indentation points are first obtained via nanoindentation experiments. A frequency histogram is then constructed and fitted to generate the overall probability density function. By deconvoluting this overall curve, the probability density distributions corresponding to individual phases can be derived. Typically, the mechanical properties of various phases in cement-based materials differ significantly: for instance, the modulus of aggregates generally exceeds 50 GPa, mortar ranges between 30 and 50 GPa, and the ITZ is usually below 30 GPa. As a result, indentation data for each phase tend to cluster in distinct regions, leading to clearly separated peaks with slight overlap in the deconvoluted probability density curves [39]. The fundamental principles of this deconvolution process are detailed below.

Studies have shown [40,41] that the elastic modulus (or hardness) of each phase in cement-based materials follows a Gaussian distribution, with the corresponding probability density function expressed as follows:

(4) $$p_{i}\left(x\right)=\frac{1}{\sqrt{2\pi \sigma_{i}^{2}}}e^{-\frac{{\left(x-\mu_{i}\right)}^{2}}{2\sigma_{i}^{2}}}$$

where $p_{i}\left(x\right)$ denotes the probability density of variable *x* under the *i*-th Gaussian component; *x* is the random variable of interest; $\mu_{i}$ represents the mean of the *i*-th Gaussian distribution; $\sigma_{i}^{2}$ is the corresponding variance; and $\sigma_{i}$ is the standard deviation.

The overall probability density function for the elastic modulus (or hardness) of all indentation points is then the weighted sum of the probability density functions of each phase [38]:

(5) $$\left\{\begin{array}{c} P\left(x\right)=\sum_{i=1}^{n}f_{i}\cdot p_{i}\left(x\right)\\ \sum_{i=1}^{n}f_{i}=1 \end{array}\right.$$

where x is the elastic modulus (or hardness). $p_{i}\left(x\right)$ and $P\left(x\right)$ are the probability density functions of phase i and the entire material, respectively. $\mu_{i}$ and $\sigma_{i}$ are the mean and standard deviation of the elastic modulus (or hardness) of phase i. $f_{i}$ represents the fractional area under the curve $f_{i}\left(x\right)$ with respect to the total area under $f\left(x\right)$, i.e., the probability that a single indentation point falls on phase i. As previously stated, this probability is equal to the area fraction of phase i within the test region. Moreover, according to Delesse’s principle in stereology [42], the area fraction is equal to the volume fraction, so $f_{i}$ also represents the volume fraction of phase i in the test region.

In practical applications, the parameters for the elastic modulus (or hardness) distribution of each phase and their corresponding volume fractions can be obtained by fitting Equations (4) and (5) using the method of least standard deviation.

The deconvolution analysis methods are generally categorized into two types: the probability density function (PDF) method and the cumulative distribution function (CDF) method [42]. The CDF is the integral form of the PDF. Although both methods are based on similar principles, the PDF method offers greater visual clarity. Therefore, the PDF-based approach is adopted in this study for deconvolution analysis.

#### 2.2.3. Fabrication of Nanoindentation Specimens

**Figure 5:**
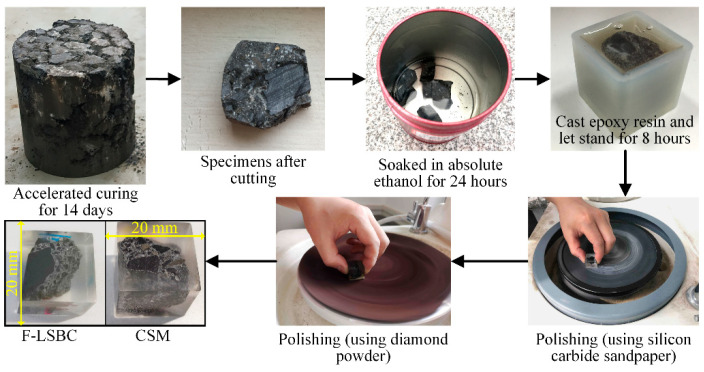
Preparation of nanoindentation specimens: F-LSBC and CSM.

The nanoindentation test was conducted in accordance with the in situ nanoindentation/scratch testing instruments for solid materials—technical specification JB/T 12721-2016 [43]. The specimen preparation process is illustrated in Figure 5.

First, cylindrical specimens cured under accelerated conditions for 14 days were cut into cubes measuring approximately 2 cm × 2 cm × 2 cm. To inhibit further hydration, the specimens were immersed in absolute ethanol for 24 h and then removed, air-dried, and placed in rubber molds with the testing surface facing downward. Epoxy resin (AB type) was slowly poured into the mold and allowed to cure at room temperature for 8 h before demolding.

Given the high surface smoothness requirements of nanoindentation testing, the cured specimens underwent systematic grinding and polishing. The grinding process was carried out sequentially using silicon carbide sandpapers of 120, 600, 1200, and 2000 grit, followed by ultrasonic cleaning in absolute ethanol for 5 min. During the polishing stage, 2.5 μm diamond powder was evenly applied to a polishing cloth, and the specimens were polished for 2 h. They were then ultrasonically cleaned again in absolute ethanol for 5 min. Finally, surface flatness was examined using an optical microscope to ensure compliance with testing standards. The resulting specimens, as shown in Figure 4, exhibited a surface finish that met the requirements for nanoindentation testing.

#### 2.2.4. Test Parameters

In this study, a NanoTest nanoindentation tester manufactured by MML (London, UK) was used, equipped with a diamond Berkovich indenter that was calibrated before each test to ensure measurement accuracy. During testing, both the loading and unloading rates were set at 0.1 mN/s, with a maximum load of 2.0 mN. Once the maximum load was reached, it was held constant for 10 s to obtain a stable indentation response [44].

**Figure 6:**
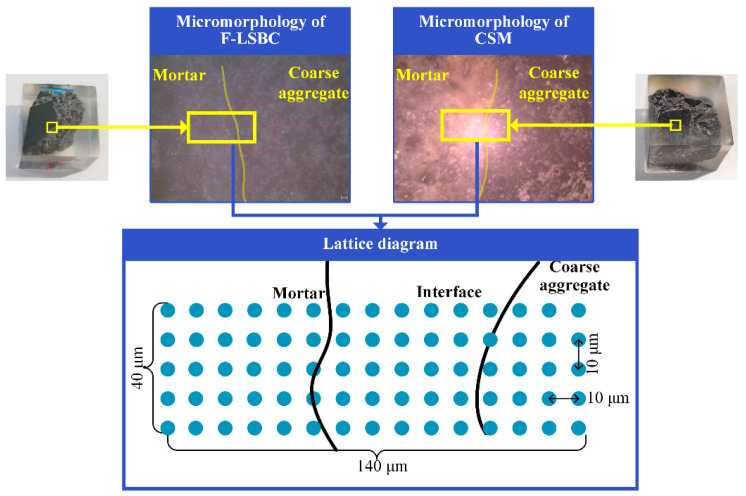
Micromorphology of F-LSBC and CSM and schematic diagram of nanoindentation test grid.

The micromorphology of the F-LSBC and CSM specimens, together with the nanoindentation test grid, is shown in Figure 6. The grid spans an area of 55 μm × 155 μm, covering coarse aggregates, the ITZ, and the surrounding mortar matrix to ensure that the characteristic heterogeneity of the composite is adequately represented. According to reference [38], the minimum spacing between adjacent indentations should be at least ten times the maximum indentation depth to avoid overlap of plastic zones. Under the applied peak load of 2.0 mN, the maximum indentation depth in cement-based materials is less than 1 μm; therefore, a spacing of 10 μm was adopted to prevent mechanical interaction between adjacent indents. In addition to meeting the mechanical separation requirement, this spacing provides sufficient spatial resolution to distinguish microscale variations across the ITZ and the bulk matrix. Accordingly, a total of 75 indentations were performed on each specimen, arranged in a 5 × 15 matrix across the selected region.

### 2.3. Macroscopic Testing

#### 2.3.1. Macroscopic Mechanical Properties Tests

This study systematically investigated the macroscopic mechanical properties of F-LSBC relative to conventional CSM materials, focusing on three key indicators: unconfined compressive strength, splitting tensile strength, and flexural strength. Cylindrical specimens were used for the unconfined compressive and splitting tensile strength tests, while flexural strength was evaluated using beam-type specimens. Three curing regimes were applied to each type of specimen: standard curing for 7 and 14 days, and accelerated curing for 14 days. For each group, five parallel specimens were prepared, and the average value was taken as the final test result. In total, 60 cylindrical specimens and 30 beam specimens were fabricated for this study. The unconfined compressive strength, splitting tensile strength, and flexural strength tests were all conducted using an MTS testing machine. The testing procedures were carried out in accordance with the relevant provisions specified in the Test Methods of Materials Stabilized with Inorganic Binders for Highway Engineering (JTG E51-2009) [32].

#### 2.3.2. Fatigue Performance Test

In accordance with the Test Methods of Materials Stabilized with Inorganic Binders for Highway Engineering (JTG E51-2009) [32], four-point bending fatigue tests on F-LSBC and CSM materials were conducted using an MTS testing machine. Given the extended fatigue testing duration, all specimens were subjected to 14-day standard curing without a 90-day aging treatment. Since the primary objective of this study was to compare the fatigue performance differences between F-LSBC and CSM, maintaining a consistent curing age for both materials was sufficient to meet the experimental requirements. The fatigue tests were performed under stress-controlled loading conditions, with a continuous Haversine waveform at 10 Hz. Based on the flexural strength of each material, four different stress ratios were selected. Due to F-LSBC’s relatively lower flexural strength, its specimens are more susceptible to early failure at high stress levels; thus, stress ratios of 0.35, 0.45, 0.50, and 0.70 were adopted. For CSM specimens, the stress ratios were set at 0.65, 0.70, 0.75, and 0.80. Three parallel specimens were tested at each stress ratio, with the ambient temperature maintained at approximately 20 °C. The test was terminated upon visible specimen failure. In addition, for specimens with longer fatigue life and extended testing durations, a moist plastic sheet was used to cover the specimen surface during loading to prevent drying and ensure stable moisture conditions, thereby maintaining test accuracy.

#### 2.3.3. Dry Shrinkage Properties Test

In accordance with the Test Methods of Materials Stabilized with Inorganic Binders for Highway Engineering (JTG E51-2009) [32], dry shrinkage tests were conducted on both F-LSBC and CSM materials. For each material, six beam-type specimens were prepared and cured under standard conditions for 7 days. Among them, three specimens were used to measure mass loss to calculate the water loss rate, while the remaining three were used to measure length change to evaluate dry shrinkage strain. To ensure consistent and clearly defined boundary conditions, the specimens used for length-change measurement were tested under free-shrinkage conditions. Specifically, both ends of each beam were left completely unrestrained, and the specimens were placed on low-friction support rollers to avoid any mechanical constraint during shrinkage. No sealing materials, clamps, or end restraints were applied, allowing the specimen to deform freely along its longitudinal direction as required by JTG E51-2009. The tests were performed in a controlled-shrinkage chamber at 20 ± 1 °C and 60 ± 5% relative humidity for 14 days. Daily measurements of specimen mass and length were recorded. Based on the standard formulas provided in the specification, the following parameters were calculated: water loss rate, shrinkage magnitude, shrinkage strain, and average shrinkage coefficient.

## 3. Results and Discussion

### 3.1. Micromechanical Properties

To ensure the reliability of the nanoindentation dataset, all indentation points underwent a two-step screening process to remove those affected by initial voids, surface defects, or material heterogeneities. First, the load–displacement (P–h) curves were examined. Indentation points exhibiting (i) abnormally steep displacement increase under low load (“soft response”), (ii) sudden pop-in or pop-out events, (iii) discontinuous or incomplete unloading segments, or (iv) irregular curve shapes inconsistent with typical viscoelastic–plastic indentation behavior were considered invalid. These features generally indicate that the indenter encountered surface voids, microcracks, or loose particles rather than compact material. Accordingly, 16 such outliers in the F-LSBC dataset and 17 in the CSM dataset were removed. Second, for the remaining points, the calculated indentation modulus was evaluated. Five CSM indentations exhibited elastic moduli above 150 GPa, far exceeding the known modulus range of limestone aggregates (typically 60–80 GPa), indicating that these indentations likely fell on unhydrated cement grains rather than the intended ITZ or matrix phase [45]. These points were also excluded. After this screening, 59 valid data points were retained for F-LSBC and 53 for CSM. This screening process ensured that the subsequent statistical nanoindentation analysis was based solely on indentation responses representative of the actual microstructural phases.

#### 3.1.1. Deconvolution Analysis

**Figure 7:**
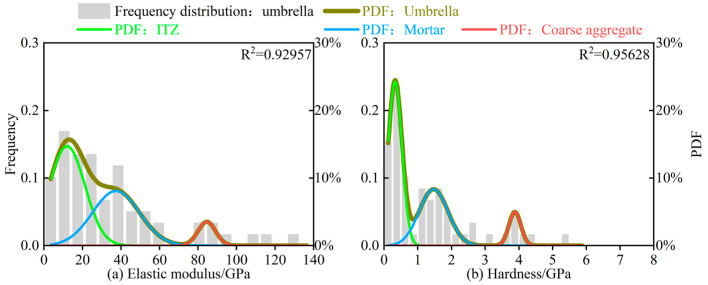
Deconvolution analysis results of nanoindentation tests for F-LSBC.

First, the overall frequency distributions of the elastic modulus and hardness of F-LSBC were fitted to obtain the overall PDF. Then, deconvolution analysis was performed on this function to derive the probability density functions corresponding to the three constituent phases. The detailed results are shown in Figure 7.

As shown in Figure 7a, the overall PDF of F-LSBC is composed of a weighted superposition of three phases, consistent with the theoretical basis described by Equation (5). Specifically, the ITZ, being the weakest region within the material, exhibits the lowest elastic modulus; the mortar phase, containing a higher concentration of cement hydration products, displays a moderate modulus, while the coarse aggregate possesses the highest modulus. Therefore, the three peaks in the probability density curve of Figure 7a, from left to right, correspond to the ITZ, mortar, and coarse aggregate, respectively. In addition, the distribution characteristics of hardness in F-LSBC exhibit the same trend as the elastic modulus, as illustrated in Figure 7b.

**Figure 8:**
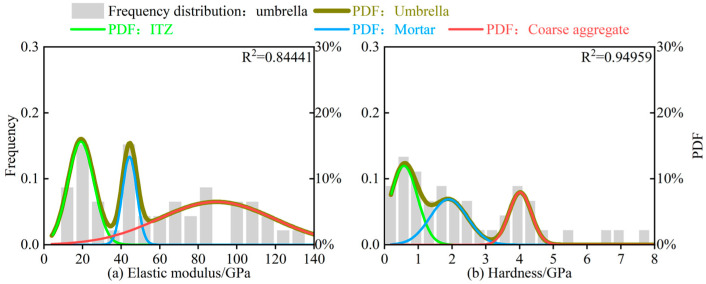
Deconvolution analysis results of nanoindentation tests for CSM.

The distribution characteristics of elastic modulus and hardness in CSM exhibit similar patterns. The detailed analysis results are presented in Figure 8.

To provide a more intuitive comparison of the elastic modulus and hardness between F-LSBC and CSM, the deconvolution analysis parameters for each phase, shown in Figure 7 and Figure 8, are summarized in Table 4.

As shown in Table 4, the elastic modulus of the coarse aggregate is 84.54 ± 3.18 GPa for F-LSBC and 89.42 ± 7.97 GPa for CSM, indicating relatively similar values. In terms of the elastic modulus of the mortar phase, F-LSBC and CSM show (37.38 ± 9.62) GPa and (44.34 ± 2.33) GPa, respectively, also reflecting a degree of similarity. However, for the ITZ, the elastic modulus of F-LSBC is (11.99 ± 3.61) GPa, whereas that of CSM reaches (19.13 ± 1.35) GPa—meaning that the ITZ modulus of F-LSBC is only about 60% of that of CSM.

Similarly, as presented in Table 4, the hardness of the coarse aggregate in F-LSBC and CSM is (3.88 ± 0.071) GPa and (4.017 ± 0.055) GPa, respectively, showing minimal difference. The hardness of the mortar phase is (1.469 ± 0.067) GPa for F-LSBC and (1.900 ± 0.168) GPa for CSM, also indicating relatively close values. However, a more pronounced difference is observed in the hardness of the ITZ: F-LSBC exhibits a hardness of (0.325 ± 0.017) GPa, whereas CSM reaches (0.576 ± 0.073) GPa—meaning that the ITZ hardness of F-LSBC is only about 55% of that of CSM.

Additionally, the volume fraction of the ITZ within the test region can be obtained by dividing the area under the ITZ probability density curve by the area under the overall probability density function. It is important to note that in the specimens used in this study, the coarse aggregate and mortar are located at the two ends of the indentation grid (see Figure 5), making their volume fractions unsuitable for direct comparison. However, the ITZ is consistently positioned at the center of the grid and remains relatively stable. Therefore, calculating and comparing the ITZ volume fractions in F-LSBC and CSM is both reasonable and representative. As shown in Table 4, the volume fraction of the ITZ in F-LSBC is 49.46% based on elastic modulus and 48.33% based on hardness, whereas the corresponding values for CSM are 30.09% and 38.27%, respectively. Overall, the ITZ volume fraction in F-LSBC is approximately 1.5 times that in CSM, indicating a higher proportion of weak phases in the F-LSBC microstructure.

#### 3.1.2. Cluster Analysis

The above deconvolution process requires manually setting the class intervals for frequency distribution, which makes the computation relatively tedious and introduces a degree of subjectivity. As observed from the probability density curves of each phase in Figure 7 and Figure 8, there are significant differences in elastic modulus (or hardness) among the coarse aggregate, ITZ, and mortar. The physical property boundaries between the three phases are relatively distinct. Therefore, it is reasonable to assume that the elastic modulus (or hardness) values of these three phases can be clearly classified into three different categories. Based on this, cluster analysis can be applied to the elastic modulus (or hardness) data of all indentation points to enable automatic identification and quantitative differentiation of the three phases. Compared with the deconvolution method, cluster analysis does not require manually defining class intervals in the frequency distribution; it only requires predefining the number of phases. As a result, it offers lower computational complexity, higher analytical efficiency, and more stable and reliable outcomes.

Clustering algorithms suitable for this analysis include K-means, hierarchical, and DBSCAN [46]. Among them, K-means clustering is the most widely used in the study of microscale mechanical properties of materials due to its high computational efficiency and ease of implementation. In this study, K-means clustering is employed for the analysis. K-means clustering evaluates the similarity between samples by calculating Euclidean distances; shorter distances indicate greater similarity and a higher likelihood of being grouped into the same category. Given the significant differences in elastic modulus and hardness among the ITZ, mortar, and coarse aggregate, K-means clustering can effectively and clearly distinguish among the phases. The final clustering results show that data within each cluster exhibit strong internal similarity, while clear distinctions are observed between different clusters. This method assumes that each data point belongs exclusively to a single category, which aligns with the fundamental assumption in statistical nanoindentation—that each indentation point represents only one material phase. This consistency ensures the logical soundness and scientific rigor of the analysis.

**Figure 9:**
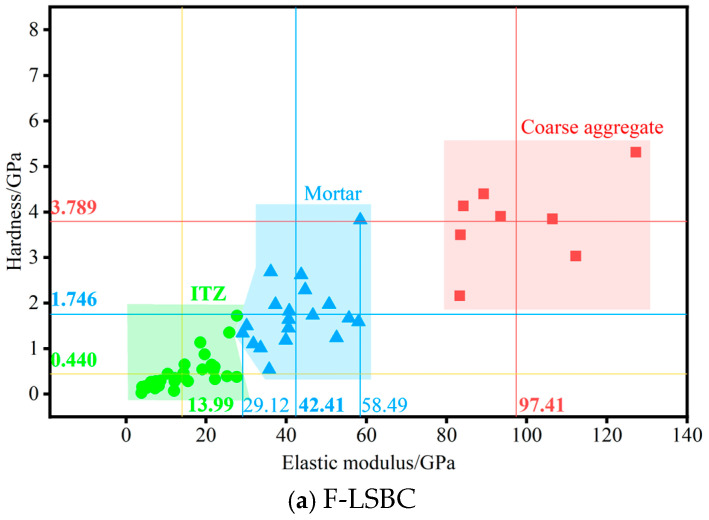

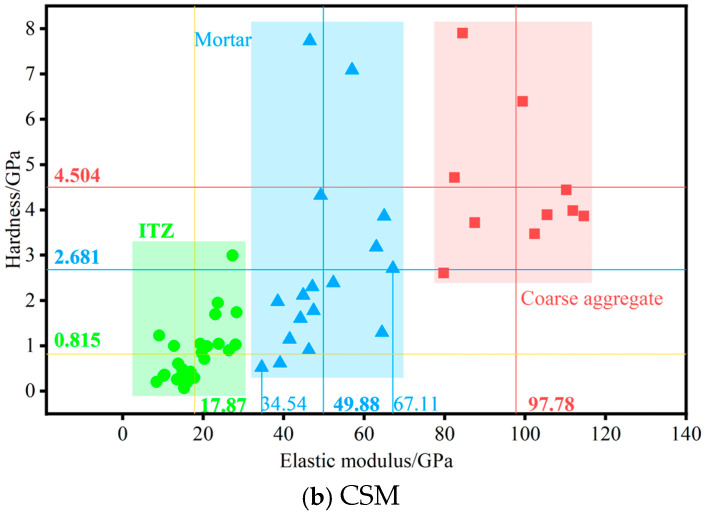
The results of cluster analysis. The bold numbers represent the average elastic modulus or hardness for each material phase (ITZ, mortar, and coarse aggregate). The regular numbers correspond to individual measurement points ob-tained from nanoindentation tests.

Before performing K-means clustering analysis, it is necessary to determine the number of clusters, K. Since this study focuses on three typical phases—coarse aggregate, ITZ, and mortar—K is set to 3. The algorithm then randomly selects three data points as the initial cluster centers and calculates the Euclidean distance from each data point to the three centers to assign each data point to the nearest cluster. The clustering process proceeds iteratively: in each iteration, the algorithm recalculates the cluster centers and reassigns data points based on the updated distances. The iteration continues until the cluster assignments stabilize—i.e., when no data points are reassigned to a different cluster. At this point, the algorithm terminates, and the final cluster centers, along with their associated data points, constitute the three identified categories. Following this procedure, the clustering results for F-LSBC and CSM are shown in Figure 9a and Figure 9b, respectively.

As shown in Figure 9a, the average elastic modulus of the ITZ, mortar, and coarse aggregate in F-LSBC are 13.99 GPa, 42.41 GPa, and 97.41 GPa, respectively—values that are highly consistent with those obtained from the deconvolution analysis. Similarly, the corresponding average hardness values are 0.440 GPa, 1.746 GPa, and 3.789 GPa, which also closely align with the deconvolution results. Figure 9b shows that the average elastic moduli of the three phases in CSM are 17.87 GPa, 49.88 GPa, and 97.98 GPa, respectively, again in good agreement with the deconvolution findings. The corresponding hardness values are 0.815 GPa, 2.681 GPa, and 4.504 GPa—slightly higher than those obtained from the deconvolution method, but still within acceptable engineering tolerance. Based on the above clustering results, a comparison of the microscale mechanical properties of the ITZ in F-LSBC and CSM reveals that the elastic modulus of F-LSBC is approximately 75% that of CSM. Although this is slightly higher than the 60% reported in the deconvolution analysis, it remains within engineering-level precision. Meanwhile, the hardness of F-LSBC is about 55% that of CSM, which is in complete agreement with the deconvolution results. This further confirms the conclusion that the ITZ in F-LSBC exhibits significantly weaker mechanical properties compared to that in CSM.

The results obtained from clustering analysis are highly consistent with those from deconvolution analysis, indicating that both methods are reasonable and effective for evaluating the microscale mechanical properties of the ITZ in F-LSBC. Moreover, each method reveals the material’s multiphase characteristics from a distinct analytical perspective. It is worth noting that clustering analysis requires only a preset number of material phases, making it straightforward to implement and computationally efficient. In contrast, deconvolution analysis requires manual selection of frequency distribution intervals, which can significantly affect the results—potentially leading to instability or convergence issues—while also involving a larger computational workload and lower overall efficiency. Therefore, given the applicability and practicality of both methods, this study recommends using clustering analysis to process and analyze nanoindentation data from multiphase materials.

#### 3.1.3. Spatial Distribution Characteristics of Elastic Modulus

In deconvolution analysis, it is assumed that the elastic modulus of each phase follows a normal distribution, and the corresponding distribution curves are obtained by fitting probability density functions. This fitting process inevitably leads to a certain degree of overlap in the modulus distributions between different phases, as shown in Figure 7 and Figure 8. In some cases, the distributions of coarse aggregate and the ITZ intersect, introducing ambiguity and complicating subsequent phase identification and quantitative analysis. In contrast, the clustering analysis method does not rely on the fitting of probability density functions. Instead, it classifies each indentation point directly based on the data’s intrinsic characteristics. As a result, the classification of elastic modulus across different phases is more distinct and shows no overlap, as illustrated in Figure 8. This clear differentiation is particularly advantageous for further investigation of the mechanical behavior of specific phases, especially for a refined analysis of weak phases such as the ITZ.

As shown in Figure 9a, the elastic modulus range for mortar in F-LSBC is 29.12–58.49 GPa. To simplify the analysis, indentation points with elastic modulus values within this range can be classified as mortar; those with values below 29.12 GPa can be assigned to the ITZ; and those exceeding 58.49 GPa can be categorized as coarse aggregate. Similarly, according to Figure 9b, the elastic modulus range for mortar in CSM is 34.54–67.11 GPa. Thus, indentation points with modulus values between 34.54 and 67.11 GPa can be considered representative of mortar; values below 34.54 GPa indicate the ITZ; and values above 67.11 GPa correspond to coarse aggregate.

It is worth noting that in previous studies on the spatial distribution characteristics of elastic modulus in cement-based materials, many researchers have adopted fixed-interval classification methods to mechanically categorize indentation points [40,41,45]. Specifically, indentation points with elastic modulus between 30–50 GPa are typically classified as mortar, those below 30 GPa as the ITZ, and those above 50 GPa as coarse aggregate. Although this approach yields numerical ranges that are generally close to the results of clustering analysis in this study, it does not adequately account for the specific modulus distribution characteristics of different material systems, thereby exhibiting a degree of arbitrariness and limitation. In contrast, the K-means clustering-based classification method used in this study enables data-driven, dynamic segmentation based on the actual elastic modulus distribution of each material. It avoids reliance on subjectively defined interval boundaries, offering a more scientific and rational approach with greater adaptability and classification accuracy.

**Figure 10:**
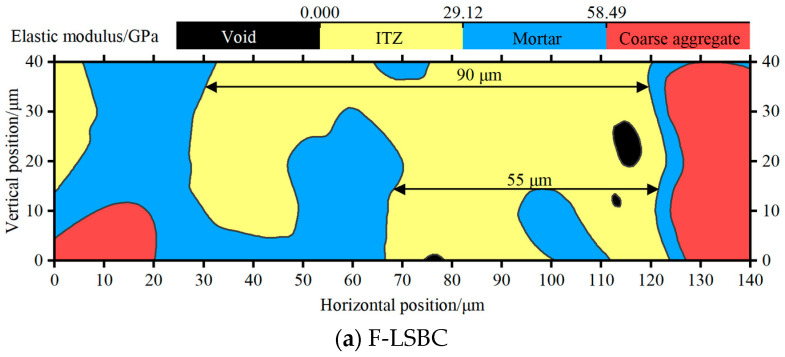

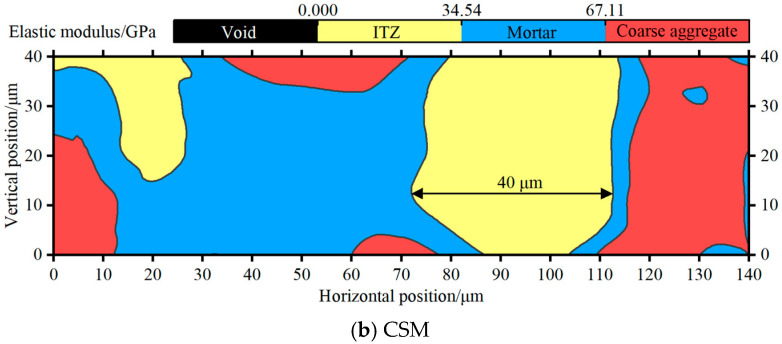
Spatial distribution characteristics of elastic modulus from nanoindentation testing.

Based on the above classification of indentation points and further incorporating the spatial coordinates of each point within the test region, spatial distribution maps of elastic modulus for F-LSBC and CSM can be constructed, as shown in Figure 10.

A comparison between Figure 10a,b reveals that the ITZ in F-LSBC occupies a significantly larger area within the test region than that in CSM. This observation is consistent with the volume-fraction results from the earlier deconvolution analysis. As shown in Table 4, the ITZ volume fraction in F-LSBC is 49.46%, whereas in CSM, it is only 30.09%. Furthermore, Figure 10a reveals the presence of relatively large voids within the ITZ of F-LSBC, most of which are located near the coarse aggregate. This suggests a weaker bond between the coarse aggregate and the surrounding matrix. This phenomenon aligns with previous research findings indicating that the porosity within the ITZ increases as it approaches the aggregate surface. Additionally, based on the spatial distribution of elastic modulus shown in Figure 10a,b, the ITZ thickness can be estimated in both materials. Figure 9a shows that the ITZ thickness in F-LSBC is approximately 55–90 μm, whereas in CSM (Figure 9b), it is about 40 μm—noticeably smaller than in F-LSBC. Previous studies have reported that the typical ITZ thickness in cement-based materials ranges from 20 to 100 μm [47]. The thickness values obtained in this study fall within this range, with F-LSBC tending toward the upper limit and CSM toward the lower limit, further confirming that the weak phase characteristics of the ITZ are more pronounced in F-LSBC.

### 3.2. Microstructural Characteristics

In the preceding sections, the elastic modulus and hardness of the ITZ were systematically investigated through deconvolution and clustering analyses. These microscale mechanical properties are closely related to the microstructural characteristics of the ITZ, particularly its porosity. Generally, higher porosity indicates a looser ITZ structure, leading to reduced load-bearing capacity and, consequently, lower elastic modulus and hardness. A significant negative correlation exists between porosity and these mechanical properties. Based on this understanding, Reference [48] conducted a quantitative study on the relationship between elastic modulus and porosity in the ITZ of cement-based materials by combining nanoindentation testing with backscattered electron (BSE) image analysis. Building on this, the study further established theoretical correlation models between elastic modulus and porosity using the differential method and the Mori–Tanaka method, as shown in Equations (6) and (7), respectively [48]. Experimental results demonstrated that both regression models exhibit a high degree of fit, providing effective tools for further elucidating the influence of pore structure on microscale mechanical performance.

(6) $$E_{ITZ}=E_{0}\cdot {\left(1-{PORE}_{ITZ}\right)}^{2}$$

(7) $$E_{ITZ}=E_{0}\cdot \frac{{\left(1-{PORE}_{ITZ}\right)}^{2}}{1-{PORE}_{ITZ}^{2}}$$

where $E_{ITZ}$ represents the elastic modulus of the ITZ measured by nanoindentation testing, in units of GPa. $E_{0}$ represents the elastic modulus of the matrix when the porosity is 0%, which can be regarded as the elastic modulus of the mortar phase, measured in GPa. ${PORE}_{ITZ}$ represents the porosity of the interfacial transition zone (ITZ), expressed as a percentage (%).

Based on Equations (6) and (7), once the elastic modulus of the ITZ is known, the porosity of the region can be back-calculated, enabling a quantitative correlation from mechanical performance to microstructural characteristics. The specific estimation formulas are as follows:

(8) $${PORE}_{ITZ}=1-\sqrt{\frac{E_{ITZ}}{E_{0}}}$$

(9) $${PORE}_{ITZ}=\frac{1-\frac{E_{ITZ}}{E_{0}}}{1+\frac{E_{ITZ}}{E_{0}}}$$

**Figure 11:**
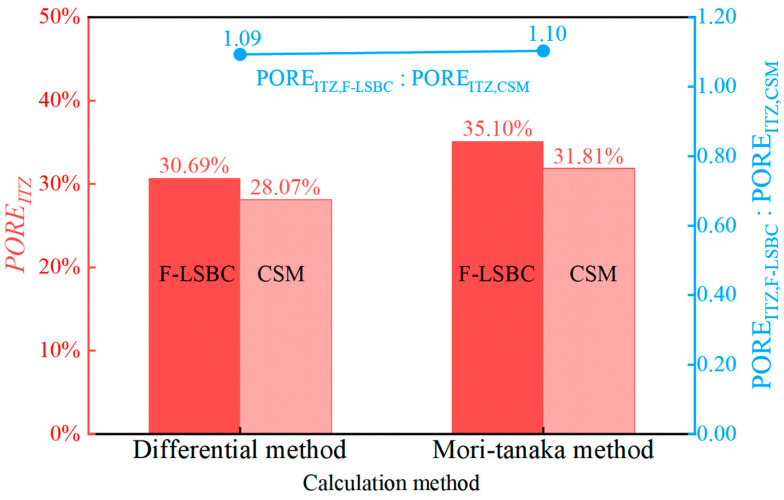
Comparison of calculated porosity in the ITZ.

According to the clustering analysis results in Figure 8, the average elastic modulus of the ITZ is 13.99 GPa for F-LSBC and 17.87 GPa for CSM; the corresponding elastic modulus of the mortar phase is 29.12 GPa for F-LSBC and 34.54 GPa for CSM. Based on these known values, the porosity of the ITZ in both materials was calculated using Equations (8) and (9). The results are presented in Figure 11.

As shown in Figure 11, based on the Differential method, the porosity of the ITZ in F-LSBC is calculated to be 30.69%, while that of CSM is 28.07%. Using the Mori–Tanaka method, the estimated porosity is 35.10% for F-LSBC and 31.81% for CSM. These results indicate that, regardless of the estimation method used, the porosity of the ITZ in F-LSBC is consistently higher than that in CSM—approximately 1.1 times greater. This finding is consistent with the previous analysis: due to the higher porosity and more porous structure of the ITZ in F-LSBC, its elastic modulus and hardness are both lower than those of CSM.

Comprehensive results from the nanoindentation tests indicate that the elastic modulus of the ITZ in F-LSBC is approximately 60–75% of that in CSM, while the hardness is about 55%. The volume fraction of the ITZ in F-LSBC is roughly 1.5 times greater than that in CSM, and its porosity is about 1.1 times higher. The ITZ thickness in F-LSBC ranges from 55 to 90 μm, compared to approximately 40 μm in CSM—collectively indicating a pronounced interfacial weakening effect in F-LSBC. The fundamental cause of this performance degradation in F-LSBC lies in its distinct mixing procedure. In F-LSBC, cement is first blended with fine aggregate to form mortar, which is then combined with coarse aggregate. This results in cement hydration products being concentrated primarily among the fine aggregates, weakening the bonding between the mortar and the coarse aggregate. Furthermore, the moisture content of the coarse aggregate in F-LSBC is controlled using a sprinkling-and-draining method without precise quantification, often leading to excess moisture. This enhances the bleeding effect in the ITZ, further increasing porosity and reducing structural compactness. Given that the ITZ around coarse aggregates is inherently a weak phase in cement-based composites, the combined effect of these factors further deteriorates its mechanical performance, resulting in a more pronounced interfacial weakening characteristic in F-LSBC.

### 3.3. Macroscopic Mechanical Properties

**Figure 12:**
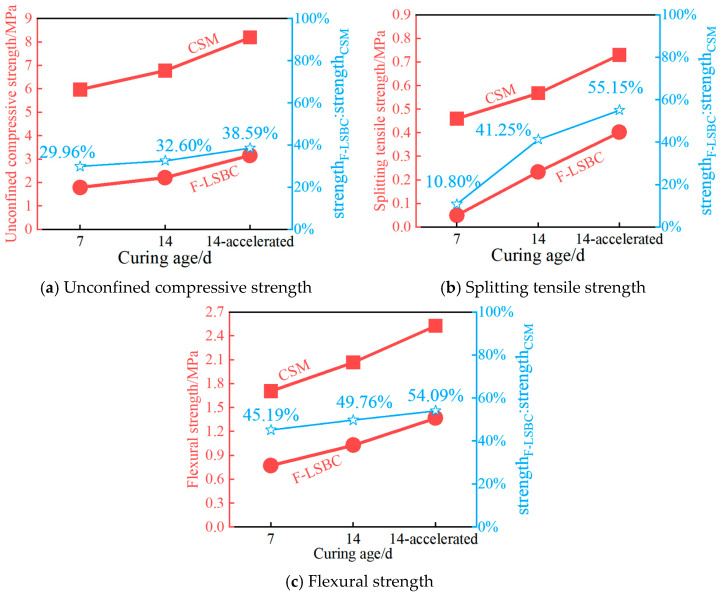
Comparison of macroscopic mechanical properties between F-LSBC and CSM.

The comparative test results of unconfined compressive strength, splitting tensile strength, and flexural strength for F-LSBC and CSM are shown in Figure 12. As observed from Figure 12, F-LSBC exhibits significantly lower values across all three mechanical strength indicators compared to CSM, indicating a substantial reduction in overall performance. This further demonstrates that the macroscopic mechanical behavior of F-LSBC is notably affected by interfacial weakening.

In F-LSBC, cement is first mixed with fine aggregate to form a filler material, which is then combined with coarse aggregate to create the final mixture. In contrast, in CSM, cement is mixed simultaneously with both coarse and fine aggregates, directly participating in the bonding of the entire aggregate skeleton. This fundamental difference in mixing procedures results in a greater concentration of cement hydration products in the fine aggregate matrix of F-LSBC, with relatively fewer products distributed at the bonding interfaces of the coarse aggregate skeleton. Given that both materials use the same cement content, the absolute amount of hydration products available for bonding coarse aggregates in F-LSBC is significantly lower than in CSM. As a result, the coarse aggregate skeleton in CSM forms a more stable and integrated structure, exhibiting superior load-bearing capacity and skeleton strength compared to F-LSBC. Considering that the coarse aggregate skeleton plays a dominant role in contributing to the compressive strength of the mixture, this structural difference directly results in a substantially lower compressive strength for F-LSBC. As shown in Figure 12a, the compressive strength of F-LSBC is less than 40% of that of CSM, clearly indicating that its overall load-bearing performance is severely compromised by weakened interfacial bonding within the aggregate structure.

In addition, under splitting and flexural loading—both governed primarily by tensile stress—the mechanical integrity of the ITZ surrounding coarse aggregates plays a decisive role in determining overall strength. Although the ITZ in F-LSBC is mechanically weaker, it is important to emphasize that, at the same curing age, the ITZ in CSM has likewise not fully matured. Consequently, the relative strength gap between the two materials is less pronounced at early ages. As shown in Figure 12b,c, the splitting tensile strength and flexural strength of F-LSBC reach approximately 40–55% of those of CSM. These comparatively higher strength ratios reflect the partially developed ITZ in both mixtures at early curing stages, mitigating the influence of interfacial weaknesses in F-LSBC. It is particularly noteworthy that, under standard 7-day curing, the overall structure of F-LSBC remains relatively loose, and interfacial bonding is extremely weak. This leads to an exceptionally low splitting tensile strength, nearly approaching 0 MPa—only 10.80% of the corresponding strength in CSM at the same age, as shown in Figure 12b. This further highlights the significant influence of interfacial development on the early-age tensile performance of cement-based materials.

As curing age increases, cement hydration progresses continuously, leading to a gradual accumulation of hydration products. Although the proportion of hydration products available for bonding the coarse aggregate skeleton in F-LSBC is relatively low, their absolute quantity still increases significantly over time. Given that the initial strength of the coarse aggregate skeleton in F-LSBC is relatively low, the increasing amount of hydration products has a more pronounced strengthening effect on its structure, resulting in a higher rate of compressive strength gain than in CSM. Specifically, as shown in Figure 12a, the compressive strength ratio of F-LSBC to CSM increases from 29.96% at 7 days of standard curing to 38.59% under 14 days of accelerated curing. Similarly, the absolute amount of hydration products distributed to the ITZ surrounding the coarse aggregates also increases with curing age. This leads to faster improvement in ITZ strength in F-LSBC, indicating that the hydration-induced enhancement of interfacial performance is more significant in F-LSBC than in CSM. This effect is reflected in the higher growth rates of splitting tensile strength and flexural strength, resulting in steadily increasing ratios of F-LSBC to CSM in these two tensile strength indicators as curing age progresses.

In summary, although F-LSBC exhibits lower unconfined compressive strength, splitting tensile strength, and flexural strength compared to CSM at all ages, it shows greater potential for mechanical performance improvement. However, due to its relatively low absolute strength levels, careful consideration must be given to the compatibility and load-bearing coordination of the entire structural system when using F-LSBC as a base material in pavement design.

### 3.4. Fatigue Performance

Following the methodology described in Section 2.3.2, fatigue tests were conducted on both F-LSBC and CSM to obtain fatigue-life data under different stress-ratio conditions. For each material, the average fatigue life was first calculated for each stress ratio. Subsequently, a linear regression analysis was performed using the average fatigue life values corresponding to four different stress ratios. In the regression analysis, the stress ratio (α) was treated as the independent variable, and the logarithm of fatigue life (lgN) was used as the dependent variable to establish a linear relationship following the general form below.

(10) lgN=A−Bα

**Figure 13:**
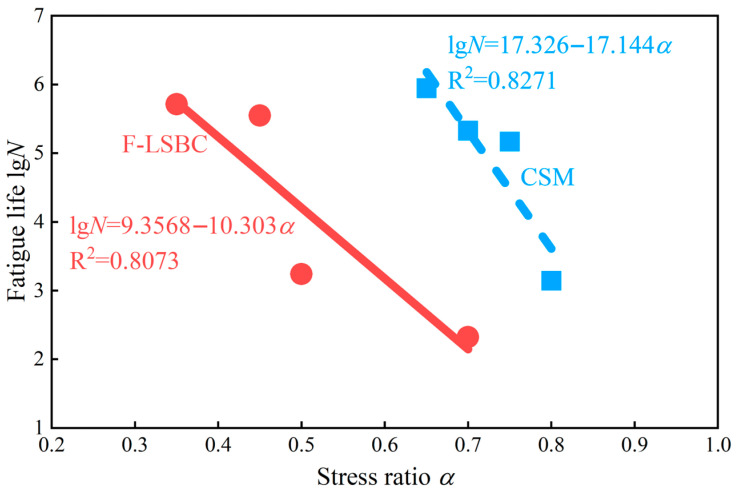
Comparison of fatigue performance between F-LSBC and CSM.

The fitted fatigue equations for F-LSBC and CSM are presented in Figure 13, with determination coefficients (R^2^) of 0.8073 and 0.8271, respectively, indicating good linear correlation for both materials. In addition, a residual analysis was conducted to assess the adequacy of the regression model. The residuals exhibited no obvious trend and were randomly distributed around zero, confirming that the assumptions of linear regression—independence, linearity, and homoscedasticity—were satisfied. These results verify that the adopted linear model is appropriate for representing the fatigue behavior of both materials under varying stress ratios.

In the fatigue equations shown in Figure 13, a larger intercept indicates a higher overall fatigue curve, meaning that the fatigue life is longer under the same stress ratio. As illustrated, F-LSBC exhibits a significantly shorter fatigue life than CSM, indicating lower fatigue resistance. In general, flexural strength plays a critical role in determining fatigue life—a lower flexural strength corresponds to a reduced ability to resist fatigue failure and results in a shorter service life [49]. As shown in Figure 12c, the flexural strength of F-LSBC is substantially lower than that of CSM, at approximately 50%. This difference is consistent with the observed fatigue life trends, further confirming the key influence of flexural strength on fatigue performance.

Fatigue failure is not an instantaneous or single-event fracture process; rather, it originates from microscopic damage concentrated in regions experiencing elevated stress or strain. This damage progressively accumulates under cyclic loading until it evolves into macroscopic failure. In cement-treated aggregates, the ITZ between coarse aggregates and the cement mortar represents the primary mechanical weak link. Its inherent vulnerability makes it the preferred site for microcrack initiation and a key factor governing fatigue damage evolution. Relative to conventional CSM, the ITZ surrounding coarse aggregates in F-LSBC presents a noticeably looser microstructure and inferior mechanical performance. As shown in Figure 7, Figure 8, Figure 9, Figure 10 and Figure 11, the elastic modulus of the ITZ in F-LSBC reaches only 60–80% of that in CSM, while its hardness is merely 55%. Additionally, the ITZ thickness in F-LSBC ranges from 55 to 90 μm, which is substantially larger than the approximate 40 μm observed in CSM. Such reduced stiffness, decreased hardness, and increased thickness jointly imply that the interfacial region in F-LSBC is more susceptible to microcrack initiation and accelerated crack coalescence. Under fatigue loading, these characteristics facilitate the development of continuous damage pathways, ultimately leading to a markedly shorter fatigue life for F-LSBC.

### 3.5. Dry Shrinkage Properties

**Figure 14:**
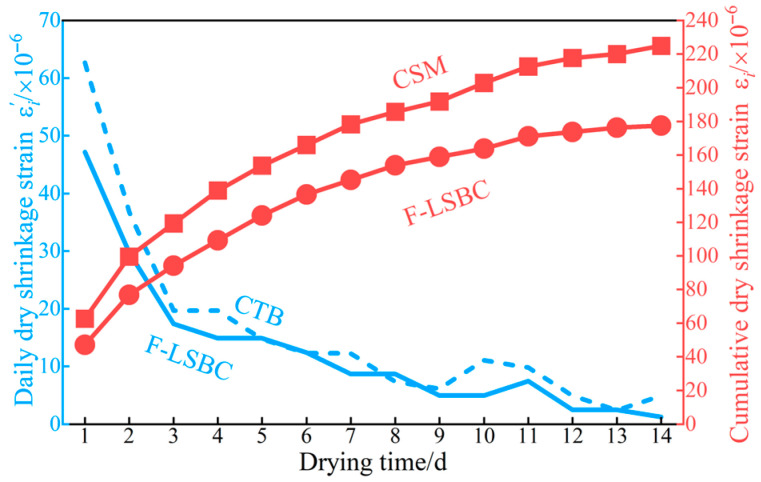
Variation in dry shrinkage strain with drying time.

The variation patterns of drying shrinkage strain with drying time for F-LSBC and CSM are shown in Figure 14. Figure 14 indicates that during the entire drying shrinkage test, the daily drying shrinkage strains $\varepsilon_{i}^{\prime }$ of both F-LSBC and CSM gradually decrease, with a diminishing rate of decline, suggesting that the shrinkage behavior of the materials progressively stabilizes. Overall, the daily drying shrinkage strain $\varepsilon_{i}^{\prime }$ exceeds 20 × 10^−6^ during the first three days; from day 4 to day 7, it ranges between 20 × 10^−6^ and 10 × 10^−6^; and after day 7, it generally falls below 10 × 10^−6^ with minimal variation, indicating that drying shrinkage has essentially stabilized. Correspondingly, the cumulative drying shrinkage strain $\varepsilon_{i}$ increases rapidly in the first three days and slows down thereafter, exhibiting a typical shrinkage evolution pattern characterized by “rapid in the early stage and slow in the later stage.”

**Figure 15:**
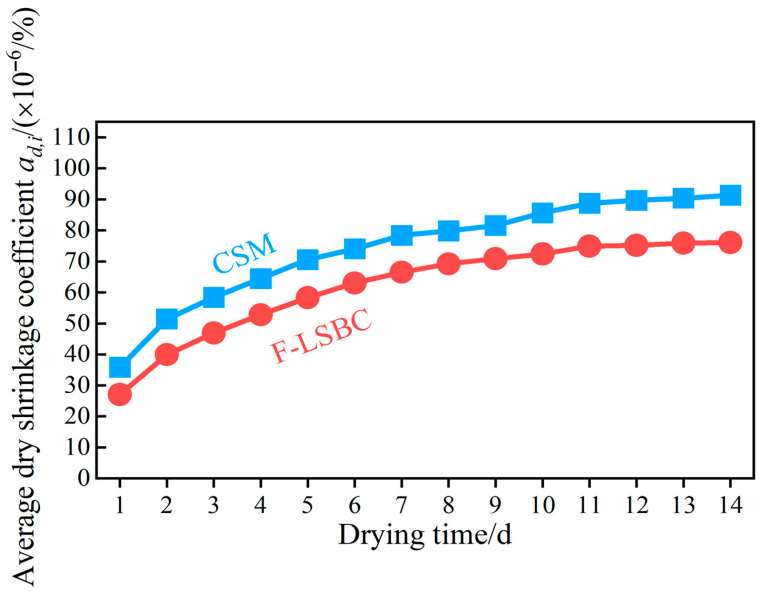
Variation in the average dry shrinkage coefficient with drying time.

Corresponding to the above trend in cumulative drying shrinkage strain $\varepsilon_{i}$, the average drying shrinkage coefficient $\alpha_{d,i}$ increases significantly during the first three days, while its growth gradually slows in the subsequent stages, as shown in Figure 15. Furthermore, as shown in Figure 14, the daily drying shrinkage strain $\varepsilon_{i}^{\prime }$ of F-LSBC is slightly lower than that of CSM throughout the drying process, resulting in a consistently lower cumulative shrinkage strain $\varepsilon_{i}$ for F-LSBC. This difference gradually increases as the drying time extends—consequently, the average drying shrinkage coefficient $\alpha_{d,i}$ of F-LSBC is also significantly lower than that of CSM, as illustrated in Figure 15, with the gap between the two likewise widening over time. These findings indicate that F-LSBC offers advantages in controlling drying shrinkage, with smaller deformation and greater stability.

Since the drying shrinkage test in this study lasted for 14 days, a regression analysis of the variation patterns of cumulative drying shrinkage strain $\varepsilon_{i}$ and the average drying shrinkage coefficient $\alpha_{d,i}$ over time is necessary to further compare the long-term shrinkage performance of F-LSBC and CSM. The detailed regression results are presented in Table 5. It is worth noting that, as indicated in the analysis of Figure 14, the shrinkage behavior gradually stabilizes after the seventh day, suggesting that the existing experimental data adequately capture the primary stages of shrinkage evolution. Therefore, performing regression fitting based on the current dataset is both reasonable and feasible.

**Figure 16:**
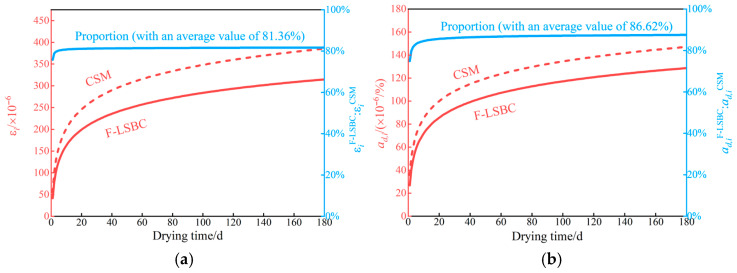
The variation pattern of drying shrinkage characteristics with drying time: (**a**) variation in cumulative dry shrinkage strain with drying time; (**b**) variation in the average dry shrinkage coefficient with drying time.

As shown in Table 5, the correlation coefficients for all four regression equations are relatively high, indicating a good fit. These equations can therefore be used with high confidence to predict the drying shrinkage characteristics of F-LSBC and CSM over an extended period (e.g., 180 days). Based on regression Equations (11) and (12), the predicted trend of cumulative drying shrinkage strain over time is presented in Figure 16a; similarly, the variation in the average drying shrinkage coefficient, derived from Equations (13) and (14), is shown in Figure 16b. From Figure 16a, the cumulative drying shrinkage strain $\varepsilon_{i}$ of F-LSBC at 180 days is approximately 81.36% of that of CSM. Figure 16b further reveals that the average drying shrinkage coefficient $\alpha_{d,i}$ of F-LSBC is about 86.62% of CSMs. These results demonstrate that F-LSBC exhibits superior resistance to drying shrinkage compared to CSM, highlighting its advantage in shrinkage control. It is important to note that due to material limitations, the CSM specimens in this study contain only 0.06% mineral powder, which is significantly lower than the 2–5% recommended by the Technical Guidelines for Construction of Highway Roadbases (JTG/T F20-2015) [50]. Previous studies have shown that higher mineral powder content leads to greater water demand and more pronounced shrinkage deformation [51]. Therefore, if CSM specimens with standard-compliant gradation were used as the reference, the shrinkage-resistance advantage of F-LSBC would be even more pronounced, with its cumulative drying shrinkage strain $\varepsilon_{i}$ and average shrinkage coefficient $\alpha_{d,i}$ further reduced compared to the control group.

In both F-LSBC and CSM, drying shrinkage primarily occurs in the cement paste. Although fine aggregates themselves do not undergo shrinkage, it is generally accepted that the mortar—composed of fine aggregates and cement paste—undergoes drying shrinkage as a whole. Coarse aggregates, on the other hand, not only do not shrink but also provide significant restraint against the shrinkage of the surrounding mortar. Therefore, from the perspective of shrinkage mechanisms, a higher volume or mass proportion of coarse aggregates results in lower voids within the aggregate skeleton, reducing the amount of shrinkable material and, consequently, the overall drying shrinkage deformation. As shown in Table 2, the mass proportion of coarse aggregates in F-LSBC is 82.44%, which is significantly higher than the 57.58% in CSM. Table 3 further reveals that the skeleton void ratio of coarse aggregates is 33.17% for F-LSBC, compared to 51.56% for CSM. These results indicate that F-LSBC contains a higher amount of coarse aggregates and possesses a denser skeleton structure. As a result, it exhibits reduced drying shrinkage deformation and superior shrinkage resistance, consistent with the experimental findings on shrinkage strain and shrinkage coefficient discussed earlier.

In addition to the lower skeleton void ratio of coarse aggregates, a more fundamental factor influencing the shrinkage behavior of F-LSBC is its relatively weak interfacial bonding between coarse aggregates and the surrounding mortar. Nanoindentation results presented earlier show that the ITZ in F-LSBC is markedly thicker (55–90 μm) than that in CSM (approximately 40 μm), while its elastic modulus reaches only 60–75% of that of CSM, and its hardness is approximately 55%. Such inferior mechanical properties render the ITZ more prone to microcrack initiation during mortar shrinkage, which weakens the aggregate–mortar bond and reduces the likelihood that aggregates will move coherently with the shrinking matrix. This weakened interface, paradoxically, restricts the transfer of shrinkage deformation into the aggregate skeleton, thereby enhancing the restraining effect of the coarse aggregates on mortar shrinkage. Consequently, the overall drying shrinkage deformation of the composite system is effectively suppressed. Therefore, despite its poorer interfacial mechanical performance, F-LSBC exhibits a “crack-without-displacement” shrinkage mechanism, which leads to reduced macroscopic shrinkage strain and further highlights its superior resistance to drying shrinkage.

**Figure 17:**
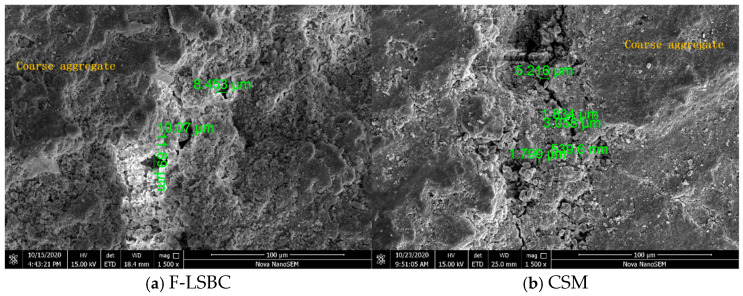
Cracking state at the coarse aggregate interface after 14 days of drying shrinkage.

To verify the above mechanistic analysis, scanning electron microscopy (SEM) was performed on F-LSBC and CSM specimens after 14 days of drying shrinkage to examine the microcrack characteristics at the ITZ between coarse aggregates and mortar. The results are shown in Figure 17a and Figure 17b, respectively. As illustrated in Figure 17a, the ITZ around the coarse aggregates in F-LSBC appears relatively loose, with clearly visible microcracks at the interface. The maximum crack width reaches 11.69 μm. These microcracks are formed as a result of mortar shrinkage being restrained by the coarse aggregate skeleton. In contrast, Figure 17b shows that the interface in CSM is denser, with fewer and narrower microcracks, the widest being 5.216 μm.

These observations suggest that, due to its higher coarse aggregate content and weaker interfacial bonding, F-LSBC tends to develop more microcracks in the ITZ during mortar shrinkage. This impedes the coordinated movement of coarse aggregates during the shrinkage process, thereby indirectly enhancing their restraining effect on the mortar. This “crack-without-displacement” mechanism inhibits the transmission and release of shrinkage strain, which ultimately manifests at the macroscopic level as lower cumulative drying shrinkage strain and average shrinkage coefficient for F-LSBC—further validating its superior shrinkage resistance.

## 4. Conclusions

This study conducted a comprehensive investigation of the macroscopic and microscopic performance of F-LSBC, with a particular focus on the ITZ around coarse aggregates. The main conclusions are as follows:

(1) Nanoindentation and statistical analysis revealed that the ITZ in F-LSBC exhibits significantly lower elastic modulus and hardness, higher porosity, and greater thickness (55–90 μm) than that in CSM. These interfacial weaknesses are the primary cause of the observed mechanical degradation.
(2) Due to ITZ deterioration, F-LSBC demonstrates markedly reduced compressive, tensile, and flexural strength, as well as shortened fatigue life, in comparison to CSM. This study quantitatively links ITZ properties with macroscopic mechanical behavior, offering a mechanistic understanding of performance loss.
(3) Despite weaker strength, F-LSBC benefits from a high coarse aggregate content and a weak interface-induced “crack-without-displacement” mechanism, which effectively reduces drying shrinkage strain and delays shrinkage development.
(4) The findings emphasize the critical role of ITZ characteristics in determining the durability and deformation behavior of cement-stabilized materials. Interface optimization is key to reconciling the strength–crack-resistance trade-off in F-LSBC, offering a pathway to improve the structural performance of semi-rigid base layers.

From an engineering perspective, the micro–macro insights obtained in this study provide useful reference points for pavement mix design. Although the ITZ of F-LSBC is relatively weaker, the overall mechanical and shrinkage performance remains adequate for field application due to its filling-type structure. This indicates that a moderately weak but uniformly distributed ITZ can be acceptable when shrinkage control is the primary design objective. The results also suggest that future mix design may balance strength and shrinkage resistance by adjusting the aggregate filling ratio and tailoring ITZ characteristics.

## Figures and Tables

**Table 1:** Physical and mechanical properties of P.O 42.5 ordinary portland cement.

Properties	Density/g·cm^−3^	Fineness/ m^2^·kg^−1^	Setting Time/Min		Compressive Strength/MPa		Flexural Strength/MPa	
			Initial Setting	Final Setting	3 d	28 d	3 d	28 d
Test result	3.065	345	150	240	22.6	54.5	4.8	9.1
Requirement		≥300	≥45	≤600	≥17.0	≥42.5	≥3.5	≥6.5

**Table 2:** Gradation of limestone aggregate and synthetic gradation of cement-stabilized macadam.

Category	Sieve Size /mm	The Original Gradation of the Limestone Aggregate			Synthetic Gradations	
		1#	2#	3#	CSM	F-LSBC
Aggregate gradation (passing rate, %)	26.5	100	100	100	100	100
	19	34.6	100	100	84.63	17.56
	16	10.27	100	100	78.91	17.56
	13.2	0	80.73	100	69.85	17.56
	9.5	0	33.88	100	53.69	17.56
	4.75	0	1.34	99.89	42.42	17.56
	2.36	0	0	70.54	29.63	17.56
	1.18	0	0	51.16	21.49	11.29
	0.6	0	0	33.98	14.27	7.89
	0.3	0	0	23.52	9.88	6.33
	0.15	0	0	14.37	6.04	3.96
	0.075	0	0	0.15	0.06	3.16
Mass fraction of synthetic gradation for CSM		23.50%	34.50%	42.00%	100%	/
Density/g·cm^−3^	Apparent density	2.739	2.739	2.725	2.733	/
	Bulk volume density	2.703	2.698	2.725	2.711	/
Mass fraction of mineral aggregates	Coarse aggregate	/	/	/	57.58%	82.44%
	Fine aggregate	/	/	/	42.42%	17.56%
	Fine-to-coarse aggregate ratio	/	/	/	0.734	0.213

**Table 3:** Compaction test results and volumetric indicators.

Indicators		Material Type		
		Filler	F-LSBC	CSM
Cement Content/%		30	5.27	52.7
Compaction Test Results	$\rho_{dry}^{max}$/g·cm^−3^	2.240	2.294	2.391
	$w_{OP}$/%	8.27	1.79	3.87
Volumetric indicators	${VCA}_{WCC}$/%	/	33.17	51.56
	${VV}_{WCC}$/%	/	15.11	12.31

**Table 4:** Deconvolution analysis parameters of nanoindentation data.

Materials	Statistical Indicators	Elastic Modulus/GPa			Hardness/GPa		
		ITZ	Mortar	Coarse Aggregate	ITZ	Mortar	Coarse Aggregate
F-LSBC	Mean value	11.99	37.38	84.54	0.325	1.469	3.88
	Standard deviation	3.61	9.62	3.18	0.017	0.067	0.071
	Volume fraction	49.46%			48.33%		
CSM	Mean value	19.13	44.34	89.42	0.576	1.900	4.017
	Standard deviation	1.35	2.33	7.97	0.073	0.168	0.055
	Volume fraction	30.09%			38.27%		

**Table 5:** Regression equations describing the variation patterns of cumulative drying shrinkage strain and average drying shrinkage coefficient with drying time *i*.

Dry Shrinkage Characteristics	Data Source	Materials	Regression Equation	R^2^	ID Number
$\varepsilon_{i}$	Figure 14	F-LSBC	$\varepsilon_{i}^{F\text{-}LSBC}=52.582ln\left(i\right)+41.674$	0.9941	(11)
		CSM	$\varepsilon_{i}^{CTB}=63.651ln\left(i\right)+54.996$	0.9937	(12)
$\alpha_{d,i}$	Figure 15	F-LSBC	$\alpha_{d,i}^{F\text{-}LSBC}=19.64ln\left(i\right)+26.725$	0.9934	(13)
		CSM	$\alpha_{d,i}^{CTB}=21.453ln\left(i\right)+35.7$	0.9973	(14)

## Data Availability

The original contributions presented in this study are included in the article. Further inquiries can be directed to the corresponding author.
